# Functionally Graded and Geometrically Modified Auxetic Re-Entrant Honeycombs: Experimental and Numerical Analysis

**DOI:** 10.3390/polym17111547

**Published:** 2025-06-01

**Authors:** Munise Didem Demirbas, Safa Ekrikaya, Umut Caliskan, Caglar Sevim, Mustafa Kemal Apalak

**Affiliations:** 1 Department of Mechanical Engineering, Erciyes University, Kayseri 38280, Türkiye; mddemirbas@erciyes.edu.tr (M.D.D.); apalakmk@erciyes.edu.tr (M.K.A.); 2 Welding Technology Program, Department of Mechanical and Metal Technologies, Vocational College of OSB, Kayseri University, Kayseri 38170, Türkiye; safaekrikaya@kayseri.edu.tr; 3 Graduate School of Natural and Applied Sciences, Erciyes University, Kayseri 38280, Türkiye; 4 Maicros Advanced Engineering Technologies, Erciyes Teknopark, Kayseri 38039, Türkiye; 5 Aviation Research and Application Center, Erciyes University, Kayseri 38280, Türkiye; 6 Department of Mechanical Engineering, Faculty of Engineering, Niğde Ömer Halisdemir University, Niğde 51240, Türkiye; caglar.sevim@ohu.edu.tr

**Keywords:** auxetic, metamaterial, negative Poisson’s ratio, functionally graded, additive manufacturing, 3D printing, finite element analysis, uniaxial compression

## Abstract

Auxetic re-entrant (RE) unit cell-based honeycombs exhibit a negative Poisson’s ratio (NPR) and possess a greater energy absorption capacity than conventional hexagonal honeycombs. The energy absorption capabilities of these structures can be further enhanced through design modifications. This study explores novel double-cylindrical-shell-based RE unit cell (REC) designs with negative Poisson’s ratios (NPRs), and the impact of material variations on NPR is analyzed in detail. The REC structures have two distinct geometric configurations: narrow REC (REC-N) and wide REC (REC-W). To demonstrate that these new geometries exhibit NPR behavior, samples were produced using additive manufacturing (AM) with materials including polylactic acid (PLA), acrylonitrile butadiene styrene (ABS), and functionally graded (FG) PLA-ABS composites. Compression tests were conducted on the samples, following ASTM-D695-15 standards, to determine the Poisson’s ratios. The experimental results obtained were validated against numerical results for all material combinations. It is demonstrated that the NPR can vary by up to 20% with changes in the REC cell geometry design for the same material combination. It is stated that changes in the material composition can alter the NPR by up to 11%. Therefore, it is shown that both the REC cell design and material variations lead to significant changes in the NPR.

## 1. Introduction

Structures exhibiting negative Poisson’s ratio (NPR) behavior are referred to as “auxetic” materials. Professor Ken Evans introduced the term “auxetic” in 1991 [1]. Auxetic materials were first discovered in nature, such as in spongy bone [2], cow udder skin [3], and aquatic salamander skin [4]. In 1987, Lakes [5] produced the first human-made foam with a negative Poisson’s ratio (NPR) through the thermal processing of polyurethane foam, and the Poisson’s ratio was measured to be −0.7. Because of their negative Poisson’s ratio (NPR), auxetic materials have been the subject of numerous studies focusing on their superior properties, such as shear modulus [6], enhanced fracture toughness [5], improved energy absorption [7], variable stiffness [8], and lower fatigue crack propagation, compared to conventional materials [9]. Shortly, with a better understanding of the mechanical advantages of auxetic materials, their use is expected to become more widespread in various engineering applications, such as improving the mechanical properties of medical devices, protective packaging of delicate components, enhancing the performance of sports equipment [10], mitigating collisions in vehicles and aircraft, and energy absorption areas in the automotive and defense industries [11].

Composite-coated and honeycomb-core sandwich structures with positive Poisson’s ratios (PPRs) have been used as engineering materials for decades in the aerospace, marine, electronics, and automotive industries because of their high stiffness and strength-to-weight ratios, as well as their well-established manufacturing techniques [12]. Auxetic honeycomb structures with negative Poisson’s ratios (NPRs) stand out as innovative engineering metamaterials because of their higher relative stiffness and strength and more efficient energy absorption than conventional honeycombs [13]. The use of these structures in composite-coated and honeycomb-core sandwich structures is currently being investigated. The literature covers auxetic topologies, including re-entrant (RE) cells, star-shaped porous cells with microstructural connectivity, chiral structures, and star arrowhead-shaped cells [14,15,16,17,18]. The literature also covers new cell geometries with negative Poisson’s ratios (NPRs) based on the re-entrant cell and its modifications.

Furthermore, recent advancements in 3D printing technologies have overcome the obstacles in producing auxetic cell structures [19,20]. As a result, the production of auxetic materials through additive manufacturing (AM) has gained momentum, and several studies on this topic have been conducted. Some of these studies are detailed below.

Ingrole et al. [21] proposed new structures by combining multiple auxetic topologies and highlighted that these hybrid structures significantly increased energy absorption capacity. Gao et al. [22] modified the triangular inner walls of the re-entrant (RE) cell structure by replacing them with cylindrical inner walls. They investigated the Poisson’s ratio of this structure using finite element analysis (FEA) and experimental methods. Their results indicated that compared to traditional RE auxetic materials, the Poisson’s ratio of the new REC-based auxetic material increased by 55.80%, while the tensile strength decreased by 44.02%. Qi et al. [23] replaced the triangular shell cell walls of the re-entrant (RE) honeycomb with double cylindrical arch cell walls and introduced a new REC configuration. They emphasized that the addition of cylindrical inner walls significantly enhanced the specific energy absorption capability of the REC honeycomb compared to the standard RE honeycomb. Li et al. [24] proposed a re-entrant, star-shaped, anti-chiral auxetic metamaterial and investigated its in-plane mechanical properties through experimental and numerical analyses. Their study highlighted that the node center distance, node diameter, and wall thickness significantly impacted the structure’s mechanical properties and deformation mode. Moreover, they found that, compared to other parameters, wall thickness had a more significant effect on the mechanical properties. Dong et al. [25] proposed a new RE auxetic metamaterial to improve mechanical performance compared to the traditional RE structure. They conducted experimental and numerical analyses to investigate its mechanical performance under quasi-static compression. They emphasized that the proposed structure performed well in stiffness and negative Poisson’s ratio (NPR) values. Zhu et al. [26] proposed a new geometry to overcome the challenge of increasing auxetic structures’ NPR, stiffness, and energy absorption capacity. Their study introduced an elliptical ring structure into a conventional RE cell, forming a new elliptical ring RE honeycomb (EARE) geometry, which provided additional longitudinal support without restricting lateral deformation. They reported that, compared to a traditional RE honeycomb with the same wall thickness, the Poisson’s ratio decreased by 5.19%, demonstrating a more substantial auxetic effect. Furthermore, they stated that the EARE structure exhibited higher average plateau stress and a 171.63% higher specific energy absorption. Etemadi et al. [27] worked on three newly designed REC auxetic structures (REC-S, REC-Star, and REC-Flower) to enhance the energy absorption capability compared to conventional REC structures. Their study highlighted that the newly designed structures performed better in terms of equivalent relative density (∆ρ) than traditional REC structures, with the REC-Flower exhibiting the highest specific energy absorption. Zhou et al. [28] examined the effects of geometric parameters on the NPR and engineering constants. They found that serrated links in the RE auxetic honeycomb increased stiffness while preserving the auxetic effect.

Numerous studies on functionally graded materials (FGMs) have also been conducted in the last two decades, parallel to auxetic materials. With the development and widespread adoption of additive manufacturing (AM) technologies, advancements in material processing and industrial processes have accelerated, leading to improvements in FGM manufacturing processes [29]. As a result, the challenges faced in material production have been overcome, and the mechanical behavior of FGMs has been experimentally investigated in many recent studies. Although FGMs were initially developed for thermal barrier applications, the use of this advanced material has expanded. It has also been employed to address various engineering problems, such as extreme wear and corrosion resistance applications [30]. Aerospace, automotive, and biomedical applications are some of the fields that benefit from this new material. Liu et al. [31] addressed the static bending of FGM sandwich plates with an auxetic core. They described the plate model as consisting of FGM coatings on the top and bottom, with an auxetic core in the middle. They stated that the Poisson’s ratio of the core varies for different angle parameters of the unit cell. Furthermore, their study thoroughly examined the effects of geometric parameters, the gradient index, auxetic core parameters, and boundary conditions on deflections and stresses. Fu et al. [32] created slanted sandwich structures with auxetic cores and FGM plates and conducted low-speed impact tests. They presented the effects of the sandwich structure porosity, slant index, FGM pattern, slant angle, and circular spring radius on energy absorption capacity. Li et al. [33] conducted a topological design optimization study of auxetic FGM cellular composites using the level-set method. They developed a multi-objective formulation for structural stiffness and material microstructure in auxetic behavior. Li et al. [34] investigated the post-buckling behavior of sandwich plates with auxetic FGM 3D lattice cores in another study. They emphasized that the loads of NPR core sandwich plates after compression and thermal buckling were significantly higher than their PPO 3D lattice core counterparts.

A literature review revealed that investigating both auxetic honeycomb cell designs and the impact of different material applications on mechanical behavior is an intriguing and emerging topic. Therefore, future studies require further effort in proposing and constructing new auxetic honeycomb configurations with innovative mechanisms to achieve superior mechanical performances, such as higher energy absorption capacity than traditional honeycombs. Additionally, uncovering and establishing quantitative relationships between mechanical properties and various design variables is an essential research area to aid in the design process of such materials. Moreover, the geometric designs used in our study are expected to have potential applications in sports equipment, the ballistic defense industry, and the automotive industry. In sports equipment, the goal is to benefit user ergonomics while achieving the performance typically provided by materials, like foam, rubber, etc., using a single material with design modifications [35]. In the defense industry, particularly in the field of ballistics, we can cite the European Defence Agency (EDA) project from 21 October 2022, which aims to utilize structures with a negative Poisson’s ratio (auxetic) in metallic materials for ballistic steels, leveraging new developments in this area [36]. In the automotive industry, these designs could be used in studies to improve impact absorption and reduce vehicle weight to enhance fuel efficiency. Our study aims to fill this gap in the literature by investigating two new auxetic honeycomb configurations with double cylindrical shell structures, which experimentally demonstrate negative Poisson’s ratios (NPRs), replacing the triangular shell walls in the traditional re-entrant (RE) cell structure. These new REC cells, named REC-N and REC-W, were designed in different geometries and fabricated from FGM materials, including PLA, ABS, and a PLA/ABS blend. Compression tests were performed on the produced cells according to ASTM-D695-15 standards, confirming their negative Poisson ratios. Furthermore, the REC-N and REC-W structures were numerically analyzed using ABAQUS 6.14 software. Both experimental and numerical analyses confirmed that the Poisson’s ratios are negative. The numerical results were compared with the experimental findings.

## 2. Materials and Methods

### 2.1. New REC Cell Geometries

**Figure 1:**
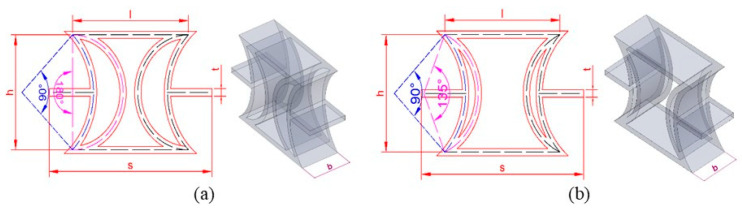
Geometric representation of the newly designed REC cells: (**a**) wide REC (REC-W) with a 90–180° double cylindrical shell; (**b**) narrow REC (REC-N) with a 90–135° double cylindrical shell.

A literature review used energy absorption capacity and negative Poisson’s ratios (NPRs) to determine the appropriate geometry. New auxetic REC cells with a double cylindrical shell structure were designed after the research. The aim was to establish relationships between independent variables in the REC cell designs, thereby ensuring the repeatability of experimental tests. The geometry of the designed auxetic REC unit cells was uniquely defined using three independent variables, i.e., the upper and lower side lengths (l), the unit cell height (h), and the unit cell width (s), based on the literature [23]. Figure 1 shows the variations in angles, thickness (t), out-of-plane width (b), l, h, and s for the newly designed structures. In Figure 1a, the narrow REC (REC-N) structure with a 90–135° double cylindrical shell is detailed, while in Figure 1b, the wide REC (REC-W) structure with a 90–180° double cylindrical shell is elaborated.

In this study, the cell geometry was determined based on manufacturability with AM and the literature, where t = 1.76 mm and b = 15 mm were selected. The dimensionless parameters used in the following equations [21] were taken as α = 0.54, β = 1.14, and γ = 0.06, and l = 32 mm, h = 28 mm, and s = 45 mm were calculated.

(1) $$\alpha =\frac{r}{h}\;\;\;(\mathrm{radius}/\mathrm{height})$$

(2) $$\beta =\frac{l}{h}\;\;\;(\mathrm{length}/\mathrm{height})$$

(3) $$\gamma =\frac{t}{h}\;\;\;(\mathrm{thickness}/\mathrm{height})$$

A patent application for the newly designed unique REC-N and REC-W geometries was submitted on 9 October 2024 through the Turkish Patent and Trademark Office Electronic Application System (epats) with application number 2024/013671.

**Figure 2:**
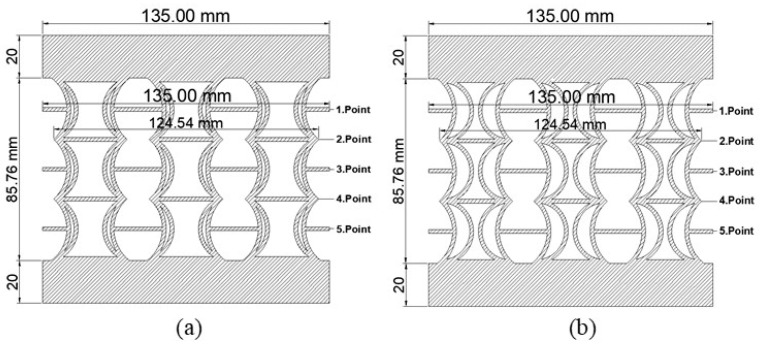
Geometric properties determined for the corresponding dimensionless parameters: (**a**) REC-N and (**b**) REC-W structures.

Figure 2a,b show the geometric properties of the REC-N and REC-W structures, respectively, for the corresponding dimensionless parameters. After the cell structures were created, rigid fillers of 20 mm were applied to the upper and lower sections for the compression test. A 3D printer produced these structures; the details are in the following section. Figure 2 shows the REC structures of the samples subjected to the compression test. Figure 2a,b show the REC-N and REC-W structures.

These structures were produced using the AM method, and the details are provided in the relevant section.

### 2.2. Production of Samples via AM

The experimental samples used in this study were fabricated using the additive manufacturing (AM) method with PLA and ABS filaments provided by Filameon [37]. PLA and ABS filaments were preferred for sample production because of their widespread use and accessibility with 3D printers, design flexibility, ease of fabrication, cost efficiency, and similar melting temperatures. The samples were produced as functionally graded (FG) structures using PLA, ABS, and PLA-ABS materials.

The technical properties of the filaments used in this study differ. PLA filaments are widely used in additive manufacturing (AM) because they can be printed in open environments and at low-temperature conditions (190–230 °C), adhere easily to the printing bed, are unaffected by ambient airflows, and do not release harmful gases during printing, making them a natural and preferred material for AM [38]. On the other hand, ABS filaments can be printed at higher temperatures (230–260 °C) and are durable and rigid. However, a heated bed is necessary to ensure proper adhesion to the printing surface. Airflows can negatively affect ABS, and rapid cooling may cause interlayer separation [39,40]. To mitigate this issue, 3D printers designed for PLA filaments can be enclosed to prevent airflows, making them suitable for printing ABS. Additionally, ABS emits harmful gases during printing. Therefore, it is recommended to ensure good ventilation or to avoid staying in the same space as the printer when printing with ABS.

**Figure 3:**
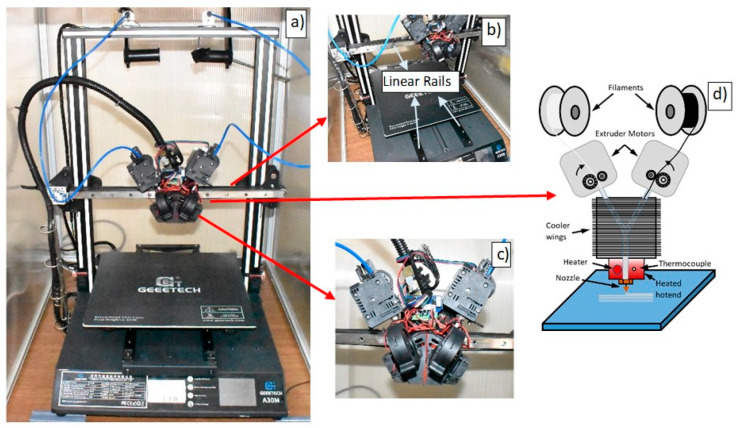
Modified 3D printer for FG production: (**a**) 2-input/1-output printer, (**b**) 2-input/1-output print head and electronic system, (**c**) general view of linear ball bearings, and (**d**) schematic representation of the 2-in-1-out printing principle.

To achieve the FG structure in additive manufacturing (AM), modified by us and branded Geeetech A30M (Shenzhen, China) a 3D printer with a dual-input and single-output print head was used (Figure 3a). This printer’s printing parameters were set to 0.2 mm layer thickness, 0.4 mm wall thickness, and 30 mm/s printing speed.

Several modifications and improvements were made to the 3D printer used to produce the FG structure and enhance its quality. To increase printing precision, reduce maintenance requirements, and eliminate vibrations during printing, the plastic wheels with bearings were removed, and linear ball bearings were installed on the axes (Figure 3b). Additionally, pushing the filaments through a long tube to the print nozzle can result in inconsistent mixing ratios during printing and retraction movements due to filament flexing. The filament feeding mechanisms were specially designed and positioned directly above the print nozzle to address this issue.

While printing FG structures in 3D printers, the design and cooling of the print head are of critical importance. ABS material requires high-temperature and enclosed conditions for printing. However, if the print head is not sufficiently cooled, PLA material can burn and cause clogging inside the nozzle during prolonged printing. To prevent this issue, the cooling fans in the 3D printer were replaced with ones providing a higher airflow (Figure 3c). Additionally, to prevent ABS material from being adversely affected by airflow, the direction of the fans’ air output was adjusted to ensure that air did not blow directly onto the printed region. This approach provided more efficient cooling and eliminated clogging issues.

The schematic representation of the 2-input, 1-output printing principle used in the 3D printer is shown in Figure 3d. After implementing all these modifications, the GCODE files for the samples to be produced were generated using Ultimaker CURA 5.5.0 software. Subsequently, the mixing ratios of the PLA and ABS filaments were adjusted by manually editing the GCODE commands. The filaments were fed to the print head via the extruder mechanism at the specified ratios defined in the GCODE. The filaments merged, melted, and were extruded in the heated print head, forming the FG structure on the bed according to the GCODE commands, thus producing the desired samples.

Samples were produced using the designed REC-N and REC-W auxetic cells arranged as three cells horizontally and vertically, with 20 mm rigid fillings added to the top and bottom sections. These samples, fabricated from ABS, PLA, and FG materials, were prepared according to ASTM-D695-15 standards and subjected to compressive loading.

**Figure 4:**
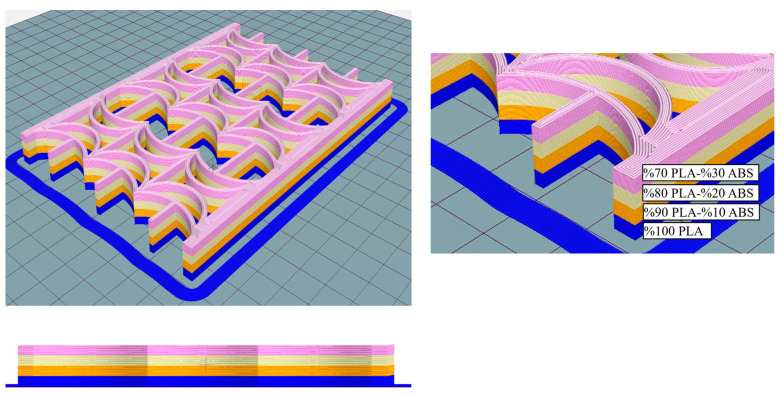
Isometric and side layer views of the FG structure with REC-W cells in GCODE format.

In another study conducted by the same research team, the compositional gradient variation was assumed to be linear for the grading of ABS and PLA materials, and FG samples were produced with 11 layers. The specified 11 layers were fabricated starting with 100% PLA, reducing PLA content by 10% and increasing ABS content by 10% in each subsequent layer, culminating in 100% ABS at the eleventh layer. However, delamination occurred in compositions containing less than 70% PLA [41]. Therefore, FG structures were produced with four layers, and the layer mixing ratios are provided in Table 1. Figure 4 shows the isometric and side views of the layers of the FG structure with REC-W cells as represented in GCODE.

### 2.3. Finite Element Model

In this study, finite element analyses (FEAs) were performed using ABAQUS 6.14 to predict the mechanical behavior of the REC-N and REC-W auxetic cellular structures under compressive loading. The base materials used in the finite element model included PLA, ABS, and functionally graded (FG) materials. For PLA and ABS, the material properties were obtained experimentally through uniaxial tensile tests following ASTM D638 standards. The Young’s modulus, Poisson’s ratio, and yield stress values obtained from these tests were directly assigned to the respective materials in the simulations. The materials were modeled using an isotropic elastic–plastic constitutive model, assuming a linear elastic behavior followed by plastic deformation.

For the FG structures, the material properties were assigned layer by layer, reflecting the varying compositions of PLA and ABS in each layer. A gradual transition of mechanical properties was defined according to the material mixing ratios specified in Table 1. The geometry of the REC structures was meshed using C3D8R solid elements (8-node linear brick elements with reduced integration). A mesh sensitivity analysis was conducted, and it was determined that a mesh size of approximately 110,000 elements provided a balance between computational efficiency and accuracy. Finer meshes were applied in regions with high stress concentrations, such as the curved and joint sections of the cylindrical shells, while coarser meshes were used in less critical regions.

The boundary conditions were designed to replicate the experimental compression test setup. A vertical displacement of 2 mm was applied to the top surface of the structure, while the bottom surface was fixed in all degrees of freedom. These boundary conditions correspond to the elastic region observed during the experimental compression tests, ensuring a consistent comparison between numerical and experimental results. The connections between the layers in the FG specimens were defined using a tie constraint in ABAQUS, ensuring full bonding between adjacent layers. This assumption was based on the strong interlayer adhesion observed in the samples produced by additive manufacturing.

**Figure 5:**
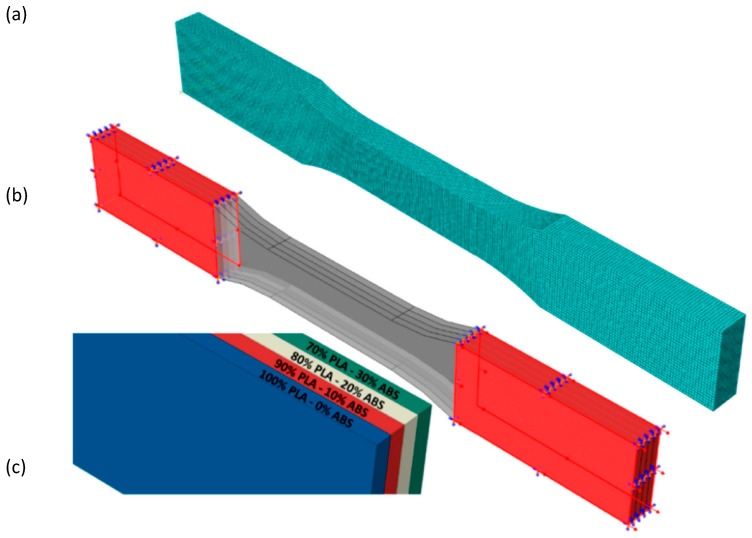
Finite element model of FG specimen: (**a**) mesh model, (**b**) boundary conditions, and (**c**) layer mix ratios [41].

While perfect bonding was assumed in the current model, potential delamination and interfacial slip effects will be considered in future studies through the implementation of cohesive zone models or contact definitions. This comprehensive approach to finite element modeling ensured that the structural response of the REC-N and REC-W auxetic cellular structures was accurately predicted, accounting for geometric, material, and loading complexities. The detailed finite element model is given in Figure 5. Analyses were performed for four different FG configurations.

## 3. Results

Through experimental and numerical analyses, this section examined the mechanical behaviors of ABS, PLA, and FG samples with the REC-N and REC-W cells produced with specified layer thicknesses. To determine the Poisson’s ratios of the REC structures created by the 3D printer, the mechanical behavior under compressive loading was experimentally investigated, and it was found that the structures exhibited a negative Poisson’s ratio (NPR). The compressive behavior of the REC structures was also determined numerically using FEA, and the results were compared with the experimental findings.

### 3.1. Numerical Analyses of REC Structure Results

The numerical analyses of the REC-N and REC-W cellular structures, presented in Figure 2, were conducted using ABAQUS 6.14 software. Samples of the REC structures were designed with four and eight layers based on the layer thickness. The C3D8R solid element was selected in the FEA modeling, and approximately 110,000 elements were utilized. The tensile behavior of the FG structures was studied using experimental samples prepared by ASTM standards, and various mechanical tests were conducted to obtain the data required for numerical modeling. Subsequently, the REC structures were numerically modeled.

Additionally, to validate the experimental data, tensile tests were performed on single-layer samples produced at specific volume fractions according to the ASTM D638 standard, and a numerical model was created using the finite element method [41]. The mechanical behaviors and stress–strain curves of the experimental samples used in this study, based on their layer thicknesses, are provided in detail. The material properties determining the mechanical behavior of each layer were transferred to the finite element code using data obtained from the tensile tests.

**Figure 6:**
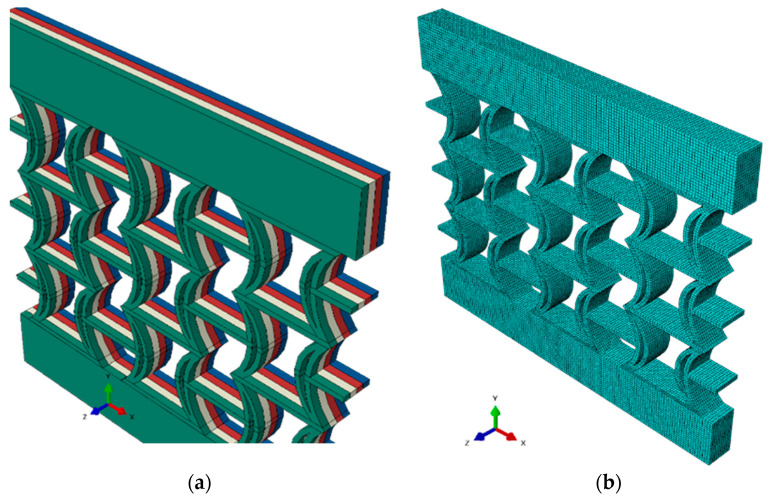
The numerical model: (**a**) FG REC-N cell structure and (**b**) its meshed version.

The experimental analyses calculated the Poisson’s ratio based on a 2 mm vertical displacement under elastic conditions. Accordingly, in the numerical analyses, a vertical displacement of 2 mm was also assumed for the modeling. Using the experimental data, the Poisson’s ratios of the REC-N and REC-W cell structures were calculated by applying the compressive load in the FEA, and it was determined that they exhibit NPR behavior. The numerically modeled form of the FG structure is shown in Figure 6a, and its meshed form is shown in Figure 6b.

**Figure 7:**
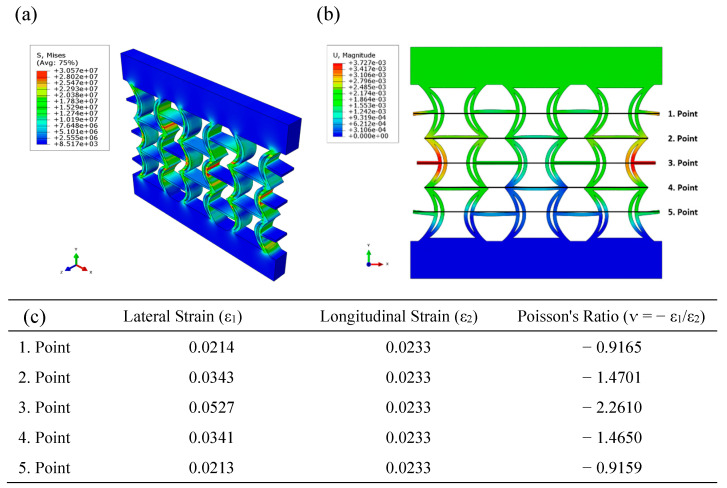
Numerical analysis results for the REC-N cellular structure made of ABS material under compressive loading: (**a**) equivalent stress distribution (Pa), (**b**) displacement distribution (m), and (**c**) Poisson’s ratios for different points.

Figure 7 illustrates the distributions of equivalent stress and displacement along the REC structure, obtained from the numerical analysis under compressive loading of the REC-N cellular structure made of ABS material. Upon examining the equivalent stress distribution, it is observed that the highest stress values are concentrated in regions near the central areas, particularly in the cylindrical sections and at the junctions where these sections connect with the horizontal struts (Figure 7a). When examining the displacement distribution, the largest displacements occur symmetrically at the outer parts of the central region (Figure 7b). Poisson’s ratios were calculated for the five points indicated in the figure, and the highest Poisson’s ratio for the REC-N structure in this material composition was obtained as −2.2610 at point 3 (Figure 7c).

**Figure 8:**
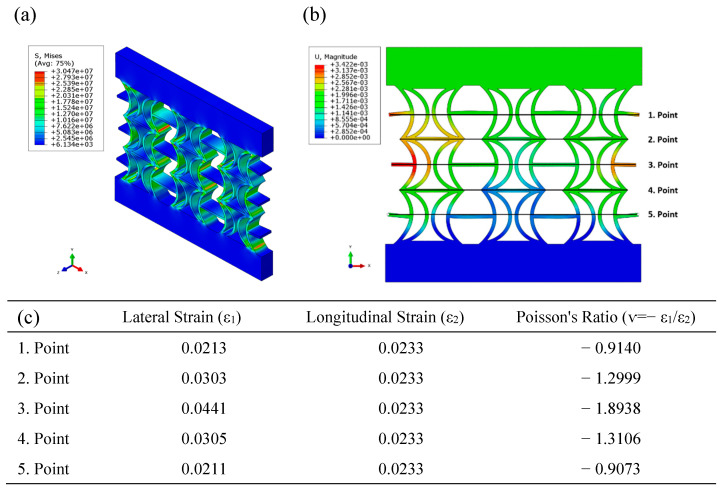
FEA results of the REC-W cellular structure made of ABS material under compressive loading: (**a**) equivalent stress (Pa) distribution, (**b**) displacement (m) distribution, and (**c**) Poisson’s ratios for different points.

Figure 8 presents the equivalent stress and displacement distributions obtained using FEA under compressive loading for the REC-W cellular structure made of ABS material. The equivalent stress distribution reveals that stress is exceptionally high near the lower and upper edges of the cylindrical sections and at the junctions where the cylindrical sections connect with the horizontal struts (Figure 8a). However, unlike the REC-N structure, the regions experiencing high-stress concentrations in the cylindrical sections are reduced (Figure 7a and Figure 8a). When examining the displacement distribution, the largest displacements are observed to occur symmetrically at the outer parts of the central region (Figure 8b). Among the Poisson’s ratios calculated for the five points indicated in the figure, the highest value was −1.8938 at point 3 (Figure 8c).

Based on the FEA results, the Poisson’s ratio of the REC-N cellular structure produced with ABS material was 19.38% higher than that of the REC-W structure.

**Figure 9:**
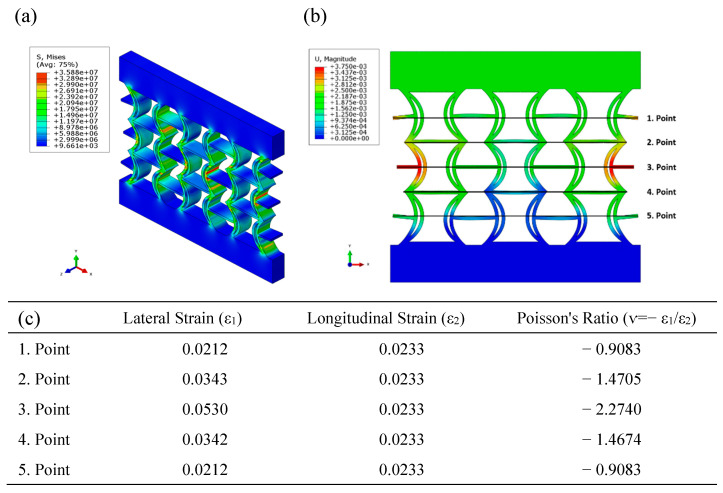
FEA results of the REC-N cellular structure made of PLA material under compressive loading: (**a**) equivalent stress (Pa) distribution, (**b**) displacement (m) distribution, and (**c**) Poisson’s ratios for different points.

Figure 9 presents the equivalent stress and displacement distributions obtained using FEA under compressive loading for the REC-N cellular structure made of PLA material. The equivalent stress distribution reveals high levels in the cylindrical sections’ central regions (Figure 9a). When examining the displacement distribution, the largest displacements are observed symmetrically at the outer parts of the central area (Figure 9b). Additionally, Poisson’s ratios were calculated for the five points indicated in the figure, and the highest Poisson’s ratio for this material composition and the REC-N structure was found to be −2.2740 at point 3 (Figure 9c).

**Figure 10:**
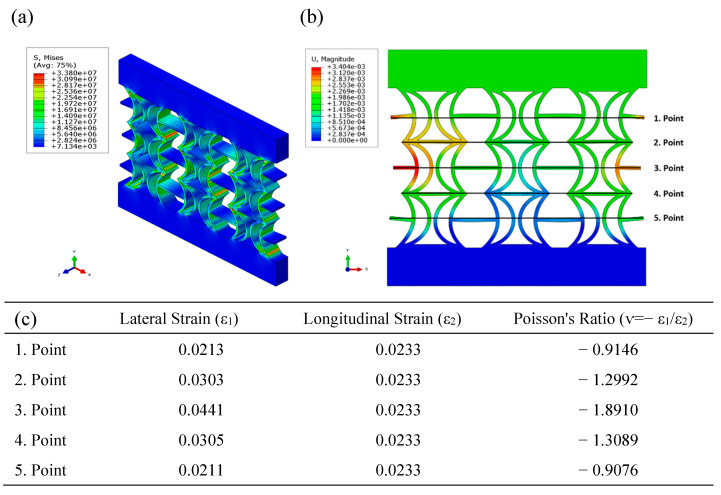
FEA results of the REC-W cellular structure made of PLA material under compressive loading: (**a**) equivalent stress (Pa) distribution, (**b**) displacement (m) distribution, and (**c**) Poisson’s ratios for different points.

Figure 10 illustrates the equivalent stress and displacement distributions obtained using FEA under compressive loading for the REC-W cellular structure made of ABS material. Examination of the equivalent stress distribution shows that the stress levels are exceptionally high along the cylindrical sections in the central regions (Figure 10a). However, compared to the REC-N structure, the areas experiencing high-stress concentrations in the cylindrical sections are reduced (Figure 9a and Figure 10a). When analyzing the displacement distribution, the most significant regions are observed asymmetrically in the outer and central parts (Figure 10b). The highest Poisson’s ratio was calculated as −1.8910, occurring at point 3 (Figure 10c) for the five points indicated in the figure. In the FEA conducted with ABS, the Poisson’s ratio of the REC-N cellular structure was calculated to be 20.25% higher than that of the REC-W structure.

Additionally, the structures made of PLA material exhibit higher equivalent stress levels than those of ABS material. When examining the displacement values, the REC-N structures have higher and more significant negative Poisson’s ratios (NPRs) than the REC-W structures.

**Figure 11:**
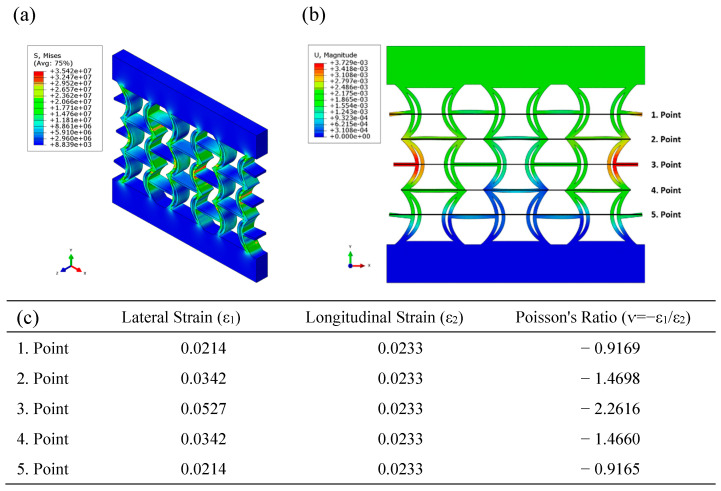
FEA results of the FG REC-N cellular structure under compressive loading: (**a**) equivalent stress (Pa) distribution, (**b**) displacement (m) distribution, and (**c**) Poisson’s ratios for different points.

Figure 11 illustrates the equivalent stress and displacement distributions obtained using FEA under compressive loading for the REC-N cellular structure made of FG materials. The equivalent stress levels are higher in the central parts of the cylindrical structures located in the middle region (Figure 11a). The regions with maximum displacement occur symmetrically in the outer areas of the central regions (Figure 11b). The highest negative Poisson’s ratio (NPR) in this region was calculated as −2.2616 (Figure 11c).

**Figure 12:**
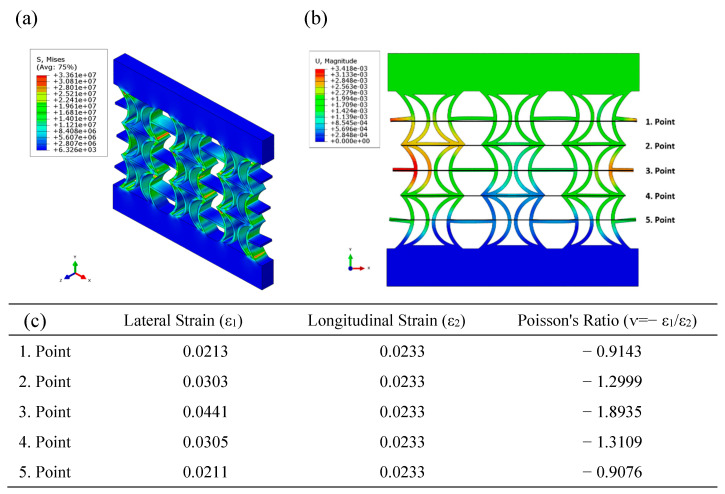
FEA results of the FG REC-W cellular structure under compressive loading: (**a**) equivalent stress (Pa) distribution, (**b**) displacement (m) distribution, and (**c**) Poisson’s ratios for different points.

Figure 12 shows the equivalent stress and displacement distributions obtained using FEA under compressive loading for the REC-W structure made of FG materials. Higher equivalent stress levels are observed in the cylindrical structures near the central regions (Figure 12a). The maximum displacement levels occur at the outer parts of the central areas (Figure 12b). The highest negative Poisson’s ratio (NPR) was calculated as −1.8935 at point 3 (Figure 12c). In the FEA conducted with FG materials, the Poisson’s ratio of the REC-N cellular structure was 19.44% higher than that of the REC-W structure. This finding highlights the significant influence of the cellular structure on the NPR across all material compositions.

In the material compositions of ABS and PLA, the regions where stress and deformation have the highest levels of impact are similar across the different REC cellular structures. In the graded structures, however, changes occur in the thickness direction in the same regions. To avoid repetition, this section will only address the Poisson’s ratios and their rates of change.

Table 2 presents the highest Poisson’s ratios, equivalent stresses, and displacement levels obtained from the numerical analysis of the REC-N and REC-W structures. The highest Poisson’s ratio, equivalent stress, and displacement levels occurred in the REC-N cellular structures made of PLA, FG, and ABS materials, respectively. The lowest equivalent stress level was observed in the REC-W cellular structure made of ABS material. Additionally, the REC-N structures exhibited higher NPRs, equivalent stress, and displacement levels compared to the REC-W structures because of their geometric designs and the contact between the inner walls of the REC-W structures during the compression test. This demonstrates the significant impact of the geometric configuration on the equivalent stress and displacement levels. This suggests that adjustable mechanical properties can be achieved by developing new designs between the REC-N and REC-W cylindrical structures.

### 3.2. Experimental Analyses of REC Structure Results

**Figure 13:**
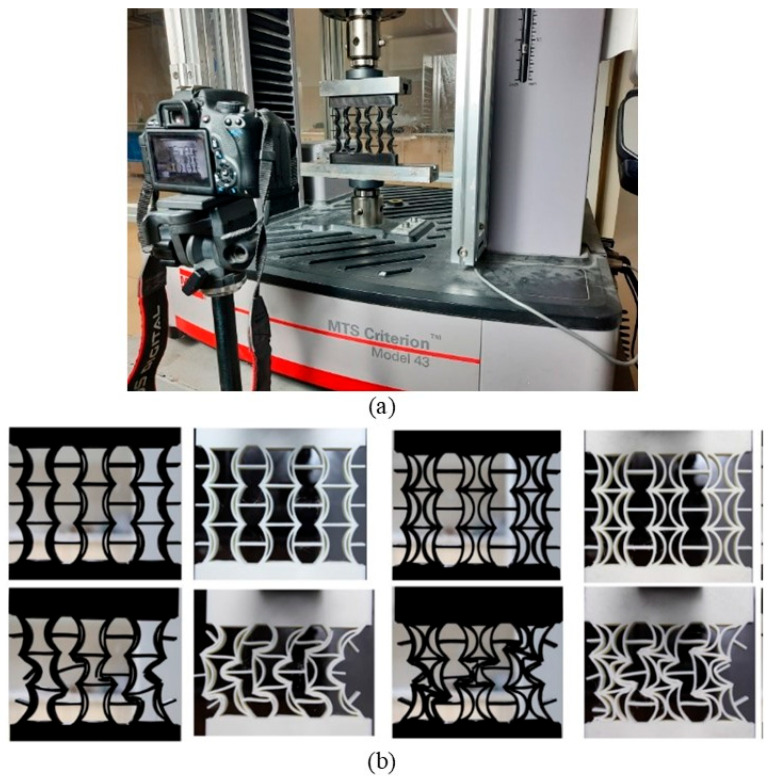
(**a**) Visual of the video recording made with a high-speed Canon camera during the compression test. (**b**) Images of the REC-N and REC-W cellular structures before and after the experimental analyses.

The compressive tests of the samples produced by the AM method using a 3D printer were performed according to the ASTM-D695-15 standards at the Erciyes University Mechanical Engineering laboratory using an MTS brand universal compression and tension testing machine [42]. High-speed video and photography were taken during the compression tests for each sample to measure their Poisson’s ratios using a Canon brand camera. The image processing method used to calculate the Poisson’s ratios was conducted using the Canon camera’s proprietary software. A visual of the compression test and the video recording during the test is shown in Figure 13a. Additionally, images of the REC-N and REC-W cellular structures before and after the experimental analyses are presented in Figure 13b.

The measurement of the NPR for the REC-N and REC-W cellular structures was based on the works of Xiao et al. [43] and Alderson et al. [16] in the literature. To calculate the Poisson’s ratios of the samples subjected to compressive loading, an elastic region corresponding to a 2 mm vertical displacement was considered. Axial and lateral contractions occurring in this region were calculated using image processing. Separate calculations were made for five points determined in the horizontal direction for the REC-N and REC-W cellular geometries produced from PLA, ABS, and FG. It was observed that the Poisson’s ratios calculated for each point of the REC-N and REC-W cellular structures for all material compositions were negative. Therefore, the structures with the newly proposed cellular geometries possess NPRs and are auxetic materials for PLA, ABS, and FG material compositions. During sample production, a 20 mm rigid fill was used at the top and bottom regions to ensure a homogeneous distribution of the compressive effect. The NPR’s absolute value was the highest at the central point (point 3).

**Figure 14:**
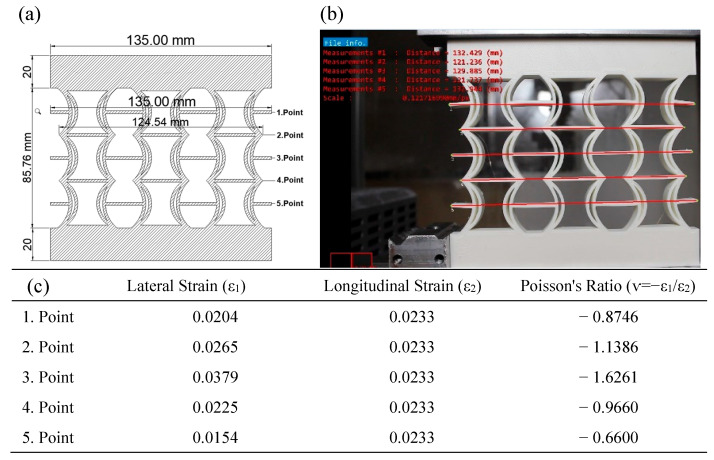
REC-N cellular structure made of ABS material under compressive load: (**a**) model view, (**b**) vertical displacement of 2 mm, and (**c**) Poisson’s ratios for different points.

In Figure 14, the Poisson’s ratios of the REC-N cellular structure produced from ABS material were calculated using the model dimensions shown in Figure 14a for five points. Then, axial and lateral measurements resulting from a 2 mm vertical displacement under compressive load (Figure 14b) were considered, and the contractions in these regions were calculated. Experimentally, the highest Poisson ratio for the REC-N structure was −1.6261 at point 3 (Figure 14c).

**Figure 15:**
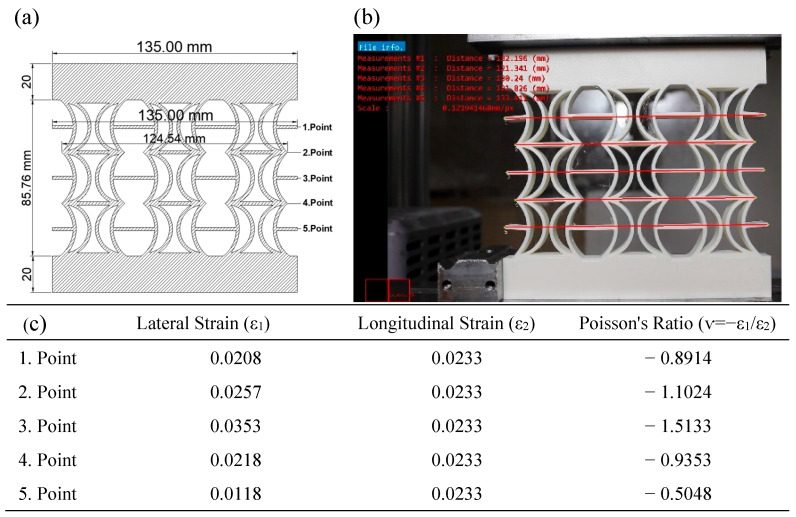
REC-W cellular structure made of ABS material under compressive load: (**a**) model view, (**b**) vertical displacement of 2 mm, and (**c**) Poisson’s ratios for different points.

In Figure 15, the Poisson’s ratios of the REC-W cellular structure made of ABS material under compressive load were calculated by considering axial and lateral contractions during a 2 mm vertical displacement. Poisson’s ratios were calculated based on the model dimensions (Figure 15a) and the measurements obtained experimentally (Figure 15b), with the highest value being −1.5133 at the third point (Figure 15c).

**Figure 16:**
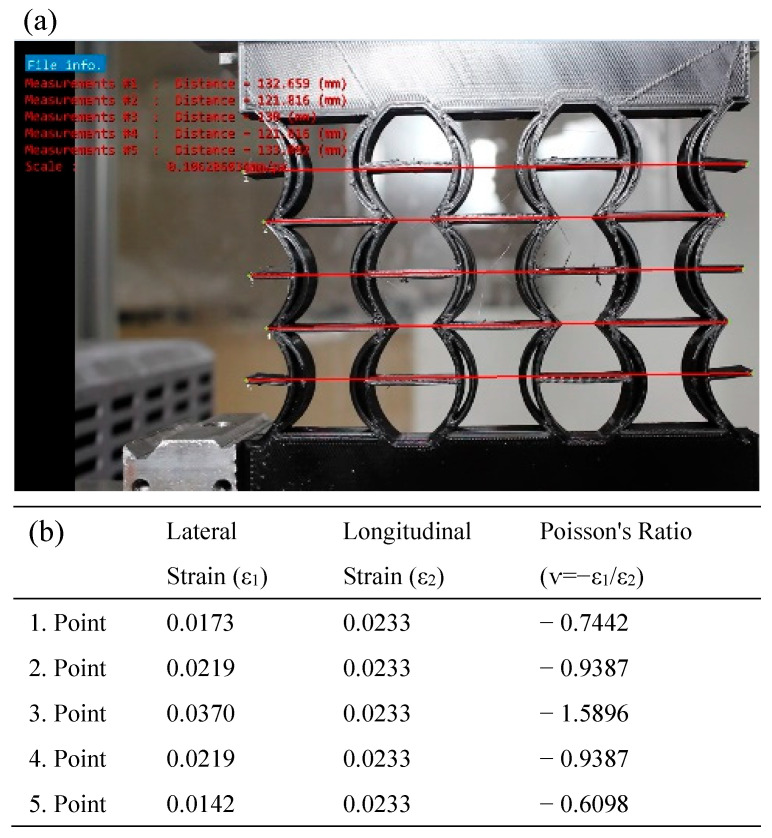
REC-N cellular structure made of PLA material under compression load: (**a**) vertical displacement of 2 mm and (**b**) Poisson’s ratios for different points.

In Figure 16, the Poisson’s ratios of the REC-N cellular structure produced from PLA material were calculated using the model dimensions shown in Figure 16a for five points. Then, axial and lateral measurements resulting from a 2 mm vertical displacement under compressive load (Figure 16b) were considered, and the contractions in these regions were calculated. Experimentally, the highest Poisson ratio for the REC-N structure was −1.5896 at point 3 (Figure 16b).

**Figure 17:**
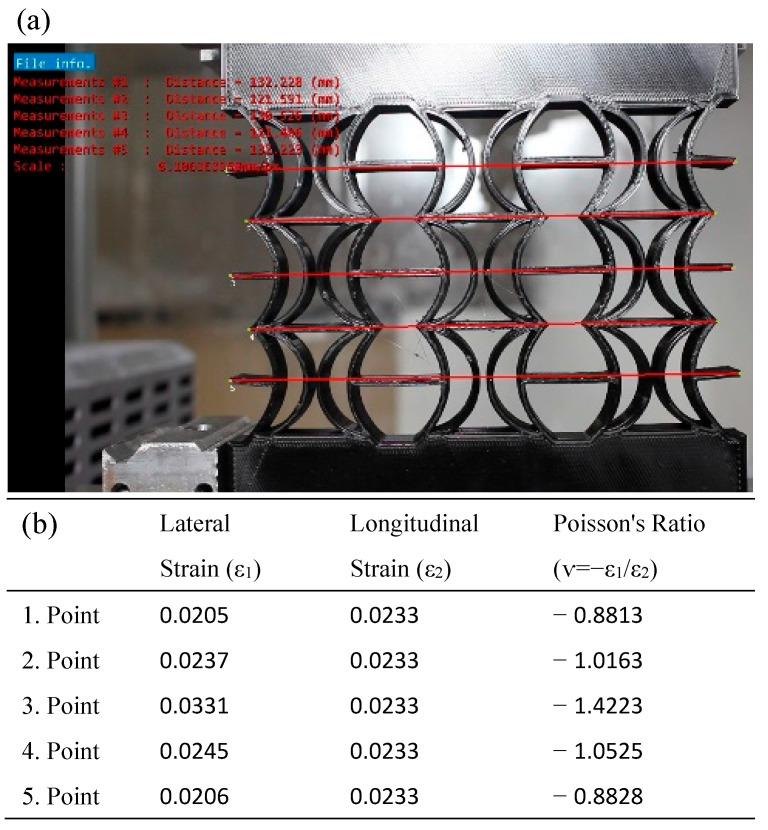
REC-W cellular structure made of PLA material under compression load: (**a**) vertical displacement of 2 mm and (**b**) Poisson’s ratios for different points.

In Figure 17, the Poisson’s ratios of the REC-W cellular structure made of PLA material were calculated under a compression load at 2 mm vertical displacement, considering axial and lateral contractions. For this REC structure, the highest Poisson ratio was −1.4223 at point 3 (Figure 17b).

**Figure 18:**
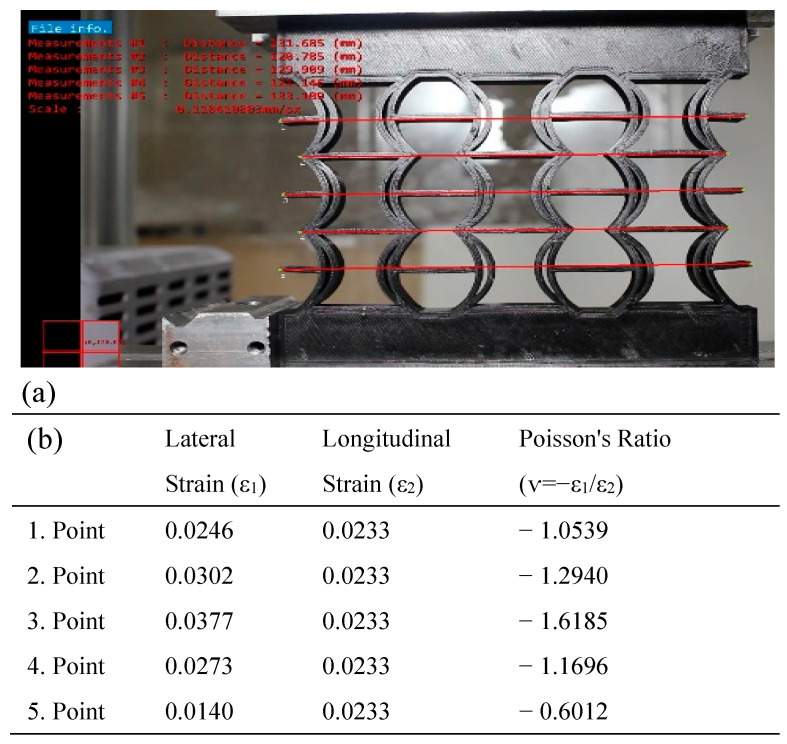
REC-N cellular structure made of FG material under compression load: (**a**) vertical displacement of 2 mm and (**b**) Poisson’s ratios for different points.

In Figure 18, the Poisson’s ratios of the REC-N cellular structure manufactured with FG material were calculated under compression loading. For this REC structure, the highest Poisson ratio was −1.6185 at point 3 (Figure 18b).

**Figure 19:**
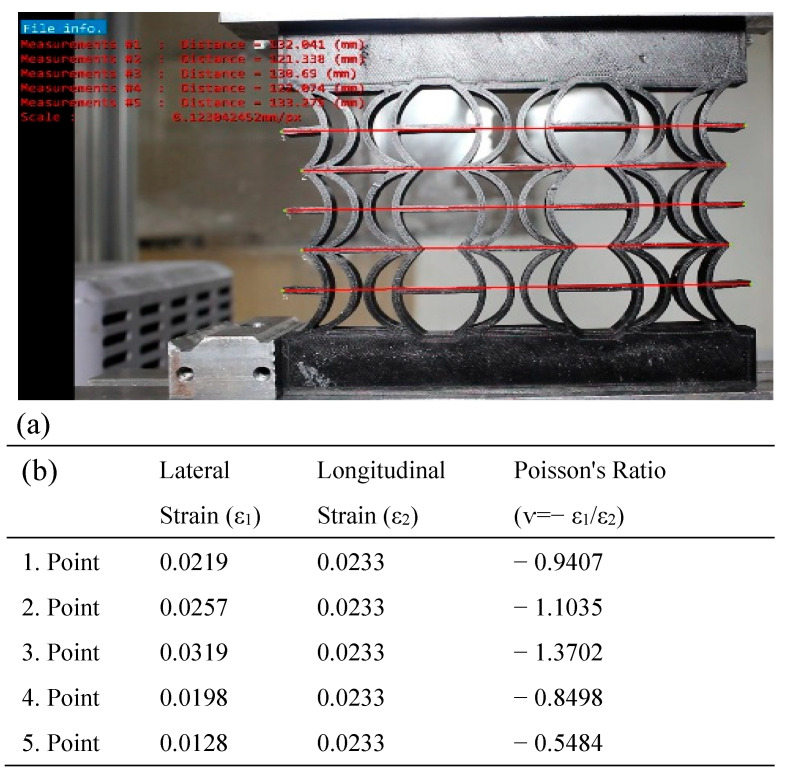
REC-W cellular structure made of FG material under compression load: (**a**) vertical displacement of 2 mm and (**b**) Poisson’s ratios for different points.

In Figure 19, the Poisson’s ratios of the REC-W cellular structure made of PLA material were calculated under compression loading for the five points shown in the model. The highest Poisson ratio was determined to be −1.3702 at point 3 (Figure 19b).

Table 3 presents the NPRs at the central points of the REC-N and REC-W structures for the ABS, PLA, and FG material compositions. In Table 3, it is shown that the Poisson’s ratio of the proposed REC-N cellular structure is higher than that of the REC-W cellular structure for all material combinations. Different modeling of the REC cell geometry with the same material composition resulted in a variation of up to 18% in the Poisson’s ratio, indicating that the REC cell structure is a significant parameter for the Poisson’s ratio. Additionally, the change in material composition altered the Poisson’s ratio by as much as 10.4%.

**Figure 20:**
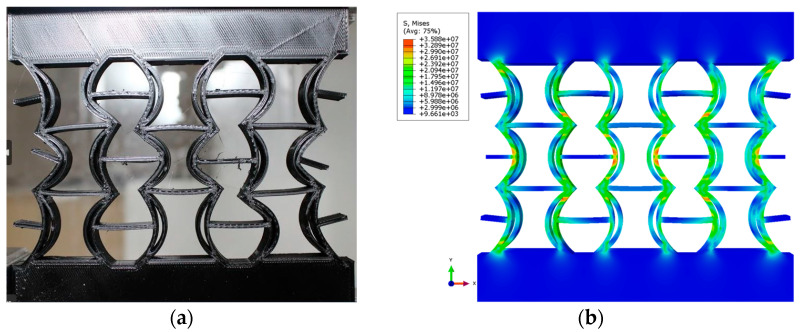
Compression behavior of the new narrow REC cellular structure made of PLA material: (**a**) experimental and (**b**) numerical compression behavior.

Table 4 presents the Poisson’s ratios at point 3, obtained experimentally and numerically, for different material combinations of the REC-N and REC-W cellular structures. The discrepancy between the numerical and experimental results for the different REC structures and materials is attributed to the more ductile behavior of the structure in the numerical model and the direct displacement of the node. However, the displacement behavior under compression loading is similar (Figure 20).

Figure 20 shows the experimental and numerical compression behavior of the new narrow REC cellular structure made of PLA material. This study’s results demonstrate that the proposed new REC structure exhibits an NPR, as confirmed by both experimental and numerical analyses. The deformation is similar in both analyses and occurs in mode X.

In this study, the Poisson’s ratios and mechanical behavior of REC-N and REC-W auxetic cell structures were investigated using both numerical and experimental methods. Both the experimental and numerical results showed that the REC-N structure has a higher negative Poisson’s ratio (NPR) compared to the REC-W structure. For example, in tests with PLA material, the Poisson’s ratio of the REC-N structure was measured experimentally as −1.5896 and numerically as −2.2740. One of the main reasons for this difference is that the material is considered isotropic in the numerical model, and microscopic defects between layers are ignored. In the experimental process, the anisotropy due to the compression direction and the differences in the bonding force between the layers caused the Poisson’s ratios to be measured differently. According to the results of the numerical analysis, the highest stress concentrations were concentrated in the curved regions of the REC cells and at the layer transition points. Experimentally, significant deformations and local fracture symptoms were observed in these regions. However, in the experiments, because of the weak bonds between the layers, local deformations occurred earlier in some regions. This resulted in lower Poisson’s ratios and different deformation modes in the experimental results.

For the FG (functionally graded) specimens, most of the differences observed between the numerical and experimental results are due to the fact that the mechanical transitions between layers were modeled in the simulation under the assumption of perfect bonding. Experimentally, the occurrence of microscopic cracks and separations at the layer transitions caused some layers to absorb less energy than expected, resulting in Poisson’s ratio deviations. The higher negative Poisson’s ratio of the REC-N structure compared to the REC-W structure indicates that this structure can provide more energy absorption. However, in the experimental tests, it was observed that some specimens showed a tendency to fracture suddenly because of the more brittle nature of the PLA material. The ABS material, on the other hand, exhibited better energy absorption, resulting in better agreement between the numerical and experimental results.

## 4. Discussion

In this study, the geometry of the proposed double cylindrical shell-based Auxetic REC unit cells with a negative Poisson’s ratio (NPR) is detailed as follows: the narrow REC (REC-N) structure with a 90–135° double cylindrical shell is illustrated in Figure 1a, and the wide REC (REC-W) structure with a 90–180° double cylindrical shell is shown in Figure 1b. In contrast, other cylindrical structures encountered in the literature include those of Gao et al. [22], who proposed a cylindrical inner wall instead of the triangular inner wall in the RE cell structure. The auxetic cylindrical inner-walled structure with an NPR and the RE structure (triangular inner wall) are shown below. Furthermore, Qi et al. [23] suggested a cylindrical shell-based design to enhance the RE metamaterial structure. In their study, they introduced a new REC configuration by replacing the inclined cell walls of the regular RE honeycomb with double circular arc cell walls. They analyzed the proposed REC cell structure’s compressive behavior theoretically and numerically.

This study’s varying cylindrical angles, instead of those in the REC-N and REC-W geometries, will directly affect the NPR. Modifying the angles between REC-N and REC-W cylinders can achieve adjustable NPRs. By conducting new studies on the dimensionless parameters used in our research, geometries with different dimensions can be developed, and the impact of these parameters on the NPR can be examined. Additionally, the REC-N and REC-W geometries, with their adjustable NPRs, have the potential to contribute to the development of features that enhance comfort and freedom of movement in ergonomic designs for wearable technologies. The defense, aerospace, and automotive industries can be adjusted according to the desired minimum energy absorption test values and employed in lightweight material applications to develop designs aimed at weight reduction and fuel efficiency optimization.

Moreover, in our study, four-layer FDM structures were produced using ABS and PLA materials, and the layer mixing ratios are provided in Table 1. By increasing the number of FDM layers, the mixing ratios of PLA and ABS can be diversified, and the effects of different FDM layers on the NPR can be investigated.

## 5. Conclusions

This study conducted experimental and numerical compression tests on two proposed REC cellular structures for ABS, PLA, and FG material compositions. These analyses revealed that the new REC-N and REC-W structures possess an NPR for all material combinations.

In both experimental and numerical analyses for all material combinations, the new REC-N cellular structure’s Poisson ratio was higher than that of the REC-W cellular structure. Different modeling of the REC cell geometry with the same material composition in the experimental method could change the Poisson’s ratio by 18%. Therefore, the REC cell structure significantly affects the Poisson’s ratio. When the REC-W cell geometry was examined, a more balanced pore distribution was found, possibly contributing to high energy absorption and stiffness.

Structures with low (or even negative) Poisson’s ratios can undergo greater deformation, allowing them to absorb more energy through plasticity and stress distribution. Both geometric designs used in our study are expected to have potential applications in sports equipment for ergonomics and cost optimization, in the defense industry for ballistic reinforcement, and in the automotive industry for impact damping and weight reduction projects.

Furthermore, in the experimental analyses, it was observed that the change in material composition led to a variation of up to 10.4% in the Poisson’s ratio. The highest Poisson ratio for the REC-N cellular structure was found with ABS materials, followed by FG and PLA materials. The Poisson’s ratio of FG-based geometries is expected to lie between those of ABS- and PLA-based geometries. Thus, different Poisson ratios can be obtained for the same geometry with FG-based structures, making it possible to adjust the NPR according to the intended application with this composition.

## Figures and Tables

**Table 1:** Material mixing ratios for each layer of the FG structure.

Layer No	PLA Ratio	ABS Ratio
Layer 1	100%	0%
Layer 2	90%	10%
Layer 3	80%	20%
Layer 4	70%	30%

**Table 2:** The highest NPR, equivalent stress (Pa), and displacement (m) levels for the REC-N and REC-W structures are based on numerical analysis results for different materials.

Material	Mechanical Properties	REC-N	REC-W
ABS	Poisson Ratio	−2.2610	−1.8938
	Equivalent Stress (MPa)	30.57	30.47
	Displacement (mm)	3.727	3.422
PLA	Poisson Ratio	−2.2740	−1.8910
	Equivalent Stress (MPa)	35.88	33.80
	Displacement (mm)	3.750	3.404
FGM	Poisson Ratio	−2.2616	−1.8935
	Equivalent Stress (MPa)	35.42	33.61
	Displacement (mm)	3.729	3.418

**Table 3:** The highest Poisson’s ratios (at point 3) of the REC-N and REC-W cellular structures.

Materials	REC-N	REC-W
ABS	−1.6261	−1.5133
PLA	−1.5896	−1.4223
FGM	−1.6185	−1.3702

**Table 4:** Experimental and numerical methods for different material combinations of the REC-N and REC-W cellular structures that obtained the highest Poisson ratios (at point 3).

Geometry	Materials	Experimental NPR	Numerical NPR	Percentage Difference
REC-N	ABS	−1.6261	−2.2610	28%
	PLA	−1.5896	−2.2740	30%
	FGM	−1.6185	−2.2616	28%
REC-W	ABS	−1.5133	−1.8938	20%
	PLA	−1.4223	−1.8910	25%
	FGM	−1.3702	−1.8935	28%

## Data Availability

The data presented in this study are available on request from the corresponding author.
